# Investigating maternal risk factors as potential targets of intervention to reduce socioeconomic inequality in small for gestational age: a population-based study

**DOI:** 10.1186/1471-2458-12-333

**Published:** 2012-06-13

**Authors:** Irene Hayward, Lorraine Halinka Malcoe, Lesley A Cleathero, Patricia A Janssen, Bruce P Lanphear, Michael V Hayes, Andre Mattman, Robert Pampalon, Scott A Venners

**Affiliations:** 1 Simon Fraser University, Faculty of Health Sciences, Burnaby, Canada; 2 University of British Columbia, School of Population and Public Health, Vancouver, Canada; 3 Child & Family Research Institute, BC Children’s Hospital, Vancouver, Canada; 4 University of Victoria, School of Public Health and Social Policy, Faculty of Human and Social Development, Victoria, Canada; 5 Department of Pathology and Laboratory Medicine, St Paul’s Hospital, Vancouver, Canada; 6 Institut National de Santé Publique du Québec, Sainte-Foy, Canada

## Abstract

**Background:**

The major aim of this study was to investigate whether maternal risk factors associated with socioeconomic status and small for gestational age (SGA) might be viable targets of interventions to reduce differential risk of SGA by socioeconomic status (socioeconomic SGA inequality) in the metropolitan area of Vancouver, Canada.

**Methods:**

This study included 59,039 live, singleton births in the Vancouver Census Metropolitan Area (Vancouver) from January 1, 2006 to September 17, 2009. To identify an indicator of socioeconomic SGA inequality, we used hierarchical logistic regression to model SGA by area-level variables from the Canadian census. We then modelled SGA by area-level average income plus established maternal risk factors for SGA and calculated population attributable SGA risk percentages (PAR%) for each variable. Associations of maternal risk factors for SGA with average income were investigated to identify those that might contribute to SGA inequality. Finally, we estimated crude reductions in the percentage and absolute differences in SGA risks between highest and lowest average income quintiles that would result if interventions on maternal risk factors successfully equalized them across income levels or eliminated them altogether.

**Results:**

Average income produced the most linear and statistically significant indicator of socioeconomic SGA inequality with 8.9% prevalence of SGA in the lowest income quintile compared to 5.6% in the highest. The adjusted PAR% of SGA for variables were: bottom four quintiles of height (51%), first birth (32%), bottom four quintiles of average income (14%), oligohydramnios (7%), underweight or hypertension, (6% each), smoking (3%) and placental disorder (1%). Shorter height, underweight and smoking during pregnancy had higher prevalence in lower income groups. Crude models assuming equalization of risk factors across income levels or elimination altogether indicated little potential change in relative socioeconomic SGA inequality and reduction in absolute SGA inequality for shorter height only.

**Conclusions:**

Our findings regarding maternal height may indicate trans-generational aetiology for socioeconomic SGA inequalities and/or that adult height influences social mobility. Conditions affecting foetal and childhood growth might be viable targets to reduce absolute socioeconomic SGA inequality in future generations, but more research is needed to determine whether such an approach is appropriate.

## Background

Differences in health status, which often occur among populations living in different socioeconomic conditions, are referred to as health inequalities. Canada’s Chief Public Health Officer described public health as “the organized efforts of society to improve health and well-being and to reduce inequalities in health [1].” Studies from around the world have reported that individuals living in areas of lower socioeconomic conditions have higher rates of adverse birth outcomes including low birthweight, small for gestational age (SGA) and preterm birth [2-12].

SGA was chosen as the birth outcome of interest here because of its association with increased morbidity and mortality throughout the life course, such as impaired cognitive function, decreased insulin sensitivity and increased risk of metabolic syndrome [13-17]. The aims of this paper are to identify a monotonic and readily accessible indicator of socioeconomic SGA inequality (bottom tenth percentile of birth weight for sex and gestational age) in the Vancouver Census Metropolitan Area (Vancouver) and to investigate whether maternal risk factors for SGA could be potential targets of intervention to reduce socioeconomic SGA inequality. Reducing socioeconomic SGA inequalities might require different public health interventions than those designed simply to reduce the overall incidence or prevalence of SGA. For example, rather than the traditional focus on individual-level risk factors, interventions designed to facilitate more equal access among socioeconomic groups to the resources and opportunities of society may be useful approaches to address socioeconomic inequality in SGA. However, because interventions targeting individual-level risk factors might also contribute to reducing SGA inequality, we investigate here whether maternal risk factors for SGA might be viable targets of intervention to reduce socioeconomic SGA inequality in Vancouver.

## Methods

### Overview

In this paper, we first used variables from the Canadian census derived at the census dissemination area (DA) level to identify one or more monotonic indicators of socioeconomic SGA inequality between DAs in Vancouver. Second, using the best indicator of inequality identified at the first step and population attributable risk percentages (PAR%), we sought to compare the magnitude of SGA risks attributable to socioeconomic inequality versus well-established individual-level maternal risk factors. Third, because different prevalence of maternal risk factors for SGA between socioeconomic groups might contribute to the SGA inequalities that we identified, we next assessed the potential of public health interventions targeting the individual-level maternal risk factors available in our study to reduce area-level socioeconomic inequality in SGA in Vancouver. To achieve the latter, we first identified which maternal risk factors were associated with increased odds of SGA in the whole Vancouver population. We then identified which of these maternal risk factors were also associated with lower area-level socioeconomic conditions. If such maternal risk factors were causally associated with both SGA and area-level socioeconomic conditions, then one could hypothesize that reducing or eliminating differences in the prevalence of those risk factors between areas of differing socioeconomic conditions would likewise reduce the SGA inequalities. Finally, we applied this thinking to investigate the potential magnitude of reductions in area-level socioeconomic SGA inequality that might result from interventions targeting individual-level maternal risk factors. We estimated the crude expected SGA risks among different socioeconomic areas assuming the prevalence of maternal risk factors in our population had a) been equal or b) reduced to zero in all areas of differing socioeconomic conditions.

### Data

We linked individual mother-child records in the British Columbia Perinatal Database Registry (the Registry) to data on socioeconomic conditions for the census DA of residence listed in the Registry at the time of birth. We chose to focus our indicator of socioeconomic conditions on measurement of material and social deprivation. Deprivation is defined as ‘a state of observable and demonstrable disadvantage relative to the local community or the wider society to which an individual, family or group belongs [18].’ We employed the deprivation index developed by the Institut national de santé publique du Québec (INSPQ) because it was defined using data at the census DA level, the smallest geographic area used in any of the available indices for our region [5,19-21]. Basing the index on a smaller geographical area should, theoretically, result in more homogenous populations within areas than would using data from larger geographical areas. However, a DA in Vancouver contains approximately 400–700 residents and so socioeconomic heterogeneity is still expected within any DA. The INSPQ deprivation index based on the 2006 Canadian census was used to obtain measures of material and social deprivation, plus their components, defined for each DA in Vancouver and ranked in quintiles.

We linked data from the INSPQ deprivation index to data from the Registry for live, singleton births in Vancouver for the period January 1, 2006 through September 17, 2009, the latest date for which complete birth data were available from hospitals at the time of our study. We chose January 1, 2006 as our starting date to correspond with the 2006 Canadian census on which the INSPQ deprivation index was based. Data on pregnancies and births came from the Registry, including clinical and demographic data that were abstracted from maternal medical records completed by physicians and midwives. The database contained records for approximately 99 percent of the births occurring during the study period in provincial health care facilities and home births [22,23].

These two data sources were linked using the postal code assigned to each DA and which appeared on the clinical records at birth. For postal codes that spanned more than one DA, a DA was randomly assigned according to the proportions of the population in the postal code living in the different overlapping areas [20]. Records with invalid or missing postal codes were excluded from the dataset (about 0.4% of records province wide, but impossible to determine which of these were in Vancouver). Perinatal Services BC (the data steward for the Registry) linked the data sources and removed all identifying information of participants prior to providing them to our study team for analysis. The study protocols were approved by the research ethics boards of Simon Fraser University (2009 s0389) and the University of British Columbia - Children’s and Women’s Health Centre of British Columbia (H09-02062) and complied with the Helsinki Declaration.

### Variables

SGA was defined as the lowest 10^th^ percentile of birthweight for gestational age and sex using a Canadian standard of infants derived from national population birthweight data (excluding Ontario) [24]. Other variables were chosen from the Registry based on their association with SGA as reported in the literature including smoking during pregnancy [15,25-27], mother’s age at delivery [6,15,26,28], parity [6,15,26,27], pre-pregnancy body mass index (BMI) [28], height [27,28], diabetes [29] and hypertension [15,28-30], and pregnancy-induced conditions (oligohydramnios [31] and placental disorders[26,29], defined using ICD-10 codes). We tested the associations of mother’s age in 5-year categories with SGA then collapsed categories after determining there were no statistically significant differences in SGA odds among categories above age 20. Height was categorized in quintiles for this analysis. Individuals with missing data for any of the above variables were excluded from the analysis.

We used the INSPQ deprivation index to define material and social deprivation, where material deprivation referred to access to modern conveniences, goods and services and social deprivation referred to relationships, customs and roles [18,20,32]. Specifically, material and social deprivation were factor scores derived from a principal component analysis carried out by INSPQ on 2006 Canadian census data for each Vancouver DA. INSPQ-derived factor scores for material and social dimensions were derived from variations of the 6 variables (and not exclusively the three most associated with each dimension) outlined in Table 1. In Vancouver, for example, the percentage of lone-parent families was closely related to both dimensions: material and social [20].

**Table 1:** Components of census dissemination area material and social deprivation in the INSPQ deprivation index

Material deprivation	Social deprivation
For persons aged 15 and over:	For persons aged 15 and over:
· Percent with no high school diploma	· Percent living alone
· Employment to population ratio	· Percent separated, divorced or widowed
· Average income	· Percent single parent families

### Statistical analysis

We first used percentages and chi-squared tests to compare those who were included to those who were in our database but excluded from the analysis. We then investigated how the information contained in the INSPQ deprivation index might best be used as an indicator of socioeconomic SGA inequality in Vancouver by analyzing the associations of SGA with 1) material and social deprivation modelled together, 2) the components of material deprivation modelled together, 3) the components of social deprivation modelled together and 4) the components of both material and social deprivation that were statistically significantly associated (alpha = 0.05) with SGA in the above analysis modelled together. We performed these analyses using hierarchical logistic regression with a random intercept for DA and an unstructured covariance structure (because individuals were clustered within DAs for which these variables were defined).

We analyzed distributions by SGA for all study variables by calculating means and percentages, and used chi-squared tests to investigate differences in the prevalence of categorical variables within levels of SGA and analysis of variance (ANOVA) to test differences in the means of height. We then developed a model of SGA including our indicator of area-level socioeconomic inequality (average income) with and without inclusion of established individual-level risk factors for SGA using hierarchical logistic regression. All variables that were statistically significantly associated with SGA in bivariate analyses at 2-sided alpha = 0.2 were initially added together to the model of SGA by average income. Individual-level variables were removed from the model by backwards elimination (according to largest *p* value) if they were not statistically significantly associated at 2-sided alpha = 0.05.

We next selected variables that were associated with increased SGA and calculated crude and adjusted population attributable risk (PAR) and PAR percentage (PAR%) for each. PAR is an estimate (under strong assumptions, which are discussed more fully in the discussion section) of the incidence of SGA in the population that would be eliminated if exposure were eliminated. The PAR% is the percentage of SGA in the population that would be eliminated if exposure were eliminated [33]. Crude PAR% was calculated by assuming the entire population had the same SGA risk as the lowest risk group of each factor (i.e. if that high-risk factor were eliminated). Adjusted PAR% was calculated using multivariate logistic regression models according to Benichou [34]. Specifically, we used odds as an approximation of risk and estimated a hierarchical logistic model with all variables included. The expected number of SGA cases was calculated by summing the individual SGA risks for all participants predicted by the model after recoding the dataset such that all individuals were in the lowest risk category of the variable of interest. Additionally, we calculated the number of cases of SGA in Vancouver that would have been avoided per year for each variable if all individuals had been in the lowest risk group at the time of our study.

PAR% gives an estimate of the magnitude change in SGA incidence that would occur if each risk factor were eliminated in the population. However, we were primarily interested in identifying potential interventions to reduce area-level socioeconomic SGA inequalities. We reasoned that if the prevalence of a maternal risk factor for SGA differed according to our indicator of area-level deprivation (higher prevalence among lower average income), then intervening to reduce and equalize the prevalence of that risk factor among levels of income might be a way to reduce the observed SGA inequality. Alternatively, if the individual-level variable associated with area deprivation was fixed and not amenable to intervention, it could at least provide a partial explanation for SGA inequality. To identify potential intervention or explanatory variables, we next investigated the association of each maternal risk factor with quintiles of area average income to determine if any were associated both with SGA and area average income. We then selected each risk factor that had higher prevalence in the lower income groups and so might be a potential intervention target or explanatory variable for socioeconomic SGA inequality. We calculated the number and percentage of SGA cases that would have been expected in each level of average income if 1) the prevalence of each risk factor had been reduced within all income levels to that found in the highest income group (i.e. the lowest SGA risk group) and 2) each risk factor had been eliminated altogether. For these analyses, we assumed that the crude SGA risks remained constant within joint levels of each variable crossed with average income quintile (for example, if a smoker in the lowest average income quintile stopped smoking, we assumed she would take on the SGA risk of a non-smoker in her income quintile). We defined absolute socioeconomic SGA inequality by subtracting the risk of SGA in the highest average income quintile from that in the lowest quintile. Relative socioeconomic SGA inequality was defined as absolute socioeconomic SGA inequality divided by the risk of SGA in the highest average income quintile [35].

Finally, we conducted a sensitivity analysis to investigate the potential for selection bias due to excluding 28% of our original sample due to missing data (mostly height and BMI). To do this, we made two comparisons of models. First, we compared the parameters in our model with and without inclusion of height and BMI (both models excluded those who were missing data on height and/or BMI). We then compared model parameters without height or BMI using included versus excluded participants. All analyses above were completed using SAS 9.2 (SAS Inc., Cary, NC).

## Results

The original linked sample contained 82,720 pregnancy and birth records from live, singleton births. Consistent with previous studies [24], we excluded births at less than 22 completed weeks gestation (n = 255), after which the remaining gestational ages at birth ranged from 22 to 43 completed weeks inclusive. We also excluded infants with birthweights less or greater than four standard deviations from mean birthweights for gestational age and sex (n = 120) using a Canadian reference [24] because these may have been the result of gestational age misclassification [24,36]. We excluded those who were missing data on pre-pregnancy BMI (n = 21,599) and average income (n = 1,707). The final sample included 59,039 live singleton births. Using chi-squared or t tests, the associations between each variable in our model (excluding BMI) and SGA were similar among those included and excluded in our analysis. The prevalence of SGA was similar in the excluded and included groups (7.4% vs. 7.2% respectively; 2-sided *p* = 0.13).

When we modelled the odds of SGA by quintiles of material and social deprivation using hierarchical logistic regression (Table 2, Model 1), the odds of SGA increased monotonically with increasing material deprivation. In contrast, none of the odds ratios of SGA between levels of social deprivation relative to the least deprived was statistically significant despite the large sample sizes. When we modelled the three material components of deprivation together (Table 2, Model 2), the odds of SGA decreased monotonically only with increasing average income. For the model of the three social components of deprivation (Table 2, Model 3), the odds of SGA increased in the third and fourth quintiles of percentage single-parent homes relative to the lowest quintile and tended towards being higher in the second and fifth quintiles. However, when we modelled average income and percentage single-parent families together (Table 2, Model 4), there was a linear negative association between SGA and average income but only slightly decreased odds of SGA in the highest quintile of single-parent families relative to the lowest quintile. Based on these results, we used average income as the indicator of within-city socioeconomic SGA inequality for the remaining analyses.

**Table 2:** Odds of SGA by census dissemination area material and social deprivation and their components

	n	OR (95% CI)
Model 1. Material and Social Deprivation (modelled together)		
Material deprivation quintile		
1 (least deprived)	9779	Reference
2	11775	1.1 (1.0, 1.3)
3	12058	1.2 (1.1, 1.4)***
4	12182	1.4 (1.2, 1.6)***
5 (most deprived)	13245	1.6 (1.4, 1.8)***
Social deprivation quintile		
1 (least deprived)	10756	Reference
2	12151	1.0 (0.8, 1.1)
3	12635	1.0 (0.9, 1.1)
4	12194	0.9 (0.8, 1.0)
5 (most deprived)	11303	0.9 (0.8, 1.1)
Model 2. Components of Material Deprivation (modelled together)		
Percent persons with no high school diploma quintile		
1 (highest percentage of diplomas)	11468	Reference
2	11718	1.0 (0.9, 1.1)
3	11752	1.0 (0.9, 1.2)
4	11995	1.0 (0.9, 1.2)
5 (lowest percentage of diplomas)	12106	1.1 (0.9, 1.3)
Employment to population ratio quintile		
1 (lowest percentage of employment)	11123	Reference
2	11635	1.0 (0.9, 1.2)
3	11927	1.0 (0.9, 1.2)
4	12120	1.0 (0.9, 1.1)
5 (highest percentage of employment)	12234	1.0 (0.9, 1.1)
Average income quintile		
1 (lowest average income)	11444	Reference
2	11510	0.9 (0.8, 1.0)
3	11953	0.9 (0.7, 1.0)**
4	12163	0.7 (0.6, 0.9)***
5 (highest average income)	11969	0.7 (0.6, 0.8)***
Percent persons living alone		
1 (lowest percentage living alone)	12206	Reference
2	12055	1.0 (0.9, 1.1)
3	11842	1.0 (0.9, 1.1)
4	11732	0.9 (0.8, 1.0)
5 (highest percentage living alone)	11204	0.9 (0.8, 1.0)
Percent persons separated, divorced or widowed		
1 (lowest percentage separated, divorced or widowed)	11759	Reference
2	11786	1.1 (1.0, 1.3)*
3	11873	1.1 (0.9, 1.2)
4	11712	1.0 (0.9, 1.1)
5 (highest percentage separated, divorced or widowed)	11909	1.0 (0.9, 1.1)
Percent single parent families		
1 (lowest percentage single parent families)	12171	Reference
2	11618	1.1 (1.0, 1.2)
3	11898	1.2 (1.0, 1.3)**
4	11606	1.2 (1.0, 1.3)**
5 (highest percentage single parent families)	11746	1.1 (1.0, 1.2)
Average income quintile		
1 (lowest average income)	11444	Reference
2	11510	0.9 (0.8, 1.0)**
3	11953	0.8 (0.7, 0.9)***
4	12163	0.7 (0.6, 0.7)***
5 (highest average income)	11969	0.6 (0.5, 0.7)***
Percent single parent families		
1 (lowest percentage single parent families)	12171	Reference
2	11618	1.1 (0.9, 1.2)
3	11898	1.1 (1.0, 1.2)
4	11606	1.0 (0.9, 1.2)
5 (highest percentage single parent families)	11746	0.9 (0.8, 1.0)*

*2-sided *p* < 0.05; ** 2-sided *p* < 0.01; *** 2-sided *p* < 0.0001.

SGA births accounted for 7.2% (n = 4,237) of all births included in our sample (Table 3). Among SGA births relative to births without SGA, there was higher prevalence of lower area-level average income, more smoking during pregnancy, fewer with maternal age ≥35, more first births, more underweight and less overweight or obese, shorter height, more hypertension, oligohydramnios, and slightly more placental disorders. Each of these associations had 2-sided *p* values <0.001.

**Table 3:** Bivariate associations of SGA with census dissemination area average income and maternal risk factors

	SGA (n = 4237)	Not SGA (n = 54802)	
n (%)	n (%)	n (%)	2-sided p*
Average income quintile			
1 ($6,196–$24,444)	1018 (24)	10426 (19)	<0.0001
2 ($24,444–$28,440)	916 (22)	10594 (19)	
3 ($28,440–$32,954)	876 (21)	11077 (20)	
4 ($32,986–$38,832)	752 (18)	11411 (21)	
5 ($38,837–$509,269)	675 (16)	11294 (21)	
Smoking during pregnancy	326 (8)	3152 (6)	<0.0001
Maternal age			
<20	75 (2)	852 (2)	0.0002
20–35	3167 (75)	39508 (72)	
≥ 35	995 (23)	14442 (26)	
Parity			
0	2784 (66)	26712 (49)	<0.0001
≥ 1	1453 (34)	28090 (51)	
Pre-pregnancy BMI (kg/m2)			
Underweight (<18.5)	531 (13)	3567 (7)	<0.0001
Normal weight (18.5–25)	2805 (66)	34913 (64)	
Overweight (25–30)	632 (15)	10862 (20)	
Obese (≥30)	269 (6)	5460 (10)	
Height (cm) (mean (SD))	161 (7)	164 (7)	<0.0001
Diabetes/hypertension			
Neither	3422 (81)	46741 (85)	<0.0001
Diabetes only	364 (9)	5251 (10)	
Hypertension only	390 (9)	2263 (4)	
Diabetes & hypertension	61 (1)	547 (1)	
Oligohydramnios	339 (8)	1044 (2)	<0.0001
Placenta disorder	135 (3)	1116 (2)	<0.0001

* Chi squared test for categorical variables and analysis of variance (ANOVA) for continuous variable (height).

**Figure 1:**
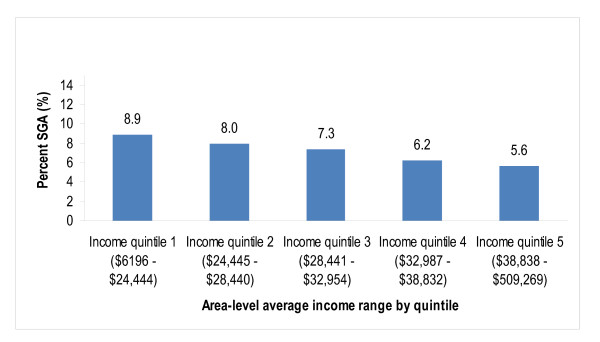
Percent SGA by census dissemination area average income quintile.

When the odds of SGA were modelled by average income alone, there was a clear monotonic association between higher average income and lower odds of SGA (Table 4 and Figure 1). The odds of SGA in the highest average income quintile were 0.6 times (*p* < 0.0001) the odds in the lowest quintile. We next added all of the maternal risk factors to this model (because all were significantly associated with SGA at 2-sided alpha = 0.2 in bivariate analyses), which attenuated the magnitudes of the associations among quintiles of average income and SGA. All maternal risk factors remained in the model at 2-sided alpha = 0.05. The odds of SGA were higher among those who smoked during pregnancy, were underweight, or had hypertension (with or without concurrent diabetes), oligohydramnios or placental disorders. The odds of SGA were lower among mothers with age <20 years, parity ≥1, or overweight or obesity and decreased with increasing maternal height.

**Table 4:** Odds of SGA by census dissemination area average income with and without adjustment for maternal risk factors

		Model 1	Model 2
Independent variables	n	OR (95% CI)	OR (95% CI)
Average income quintile			
1 ($6,196–$24,444)	11444	Reference	Reference
2 ($24,444–$28,440)	11510	0.9 (0.8, 1.0)*	0.9 (0.8, 1.0)
3 ($28,440–$32,954)	11953	0.8 (0.7, 0.9)***	0.9 (0.8, 1.0)
4 ($32,986–$38,832)	12163	0.7 (0.6, 0.8)***	0.8 (0.7, 0.9)***
5 ($38,837–$509,269)	11969	0.6 (0.5, 0.7)***	0.8 (0.7, 0.8)***
Smoking during pregnancy			
No	55561		Reference
Yes	3478		1.6 (1.4, 1.8)***
Maternal age			
<20	927		0.8 (0.6, 1.0)*
20–35	42675		Reference
≥ 35	15437		1.0 (0.9, 1.1)
Parity			
0	29296		Reference
≥ 1	29543		0.5 (0.5, 0.6)***
Pre-pregnancy BMI			
Underweight (<18.5)	4098		1.8 (1.6, 2.0)***
Normal weight (18.5–25)	37718		Reference
Overweight (25–30)	11494		0.7 (0.7, 0.8)***
Obese (≥30)	5729		0.6 (0.5, 0.6)***
Height (continuous, cm)	59039		0.9 (0.9, 0.9)***
Diabetes/ Hypertension			
Neither	50163		Reference
Diabetes only	5615		0.9 (0.8, 1.0)
Hypertension only	2653		2.4 (2.1, 2.7)***
Diabetes & hypertension	608		1.5 (1.1, 2.0)*
Oligohydramnios			
No	57656		Reference
Yes	1383		3.9 (3.4, 4.4)***
Placenta disorder			
No	57788		Reference
Yes	1251		1.4 (1.2, 1.7)**

* 2-sided *p* < 0.05; ** 2-sided *p* < 0.001; *** 2-sided *p* < 0.0001.

We calculated the crude and adjusted PAR% for variables that were associated with increased odds of SGA (Table 5). The magnitudes of crude and adjusted PAR% were similar for each variable. The largest adjusted PAR% of SGA was for the bottom four quintiles of height (51%) and first birth (32%). After these, the next largest PAR% was for the bottom four quintiles of average income (14%), which was larger than any of the remaining maternal risk factors for SGA that we investigated (PAR% from 1% to 6%).

**Table 5:** Population attributable risks for SGA of area average income and maternal risk factors

	Population attributable risk	Population attributable risk %	SGA cases/year
Crude			
Average income quintile	1.2	17.0	191
Smoking during pregnancy	0.2	2.2	22
Parity (first birth)	2.3	31.7	360
Underweight (<18.5)	0.5	6.3	69
Height (bottom four quintiles)	3.4	47.6	543
Hypertension	0.4	5.7	62
Oligohydramnios	0.4	6.1	66
Placental disorder	0.1	1.4	12
Adjusted			
Average income quintile	1.1	13.6	181
Smoking during pregnancy	0.2	2.5	34
Parity (first birth)	2.6	32.1	415
Underweight (<18.5)	0.5	6.2	89
Height (bottom four quintiles)	4.1	50.6	651
Hypertension	0.5	6.2	77
Oligohydramnios	0.6	7.4	100
Placental disorder	0.1	1.2	14

Table 6 presents the prevalence or means of maternal characteristics by average income quintiles. Among the variables that were statistically different overall in average income quintiles, several had decreased prevalence in higher average income areas including smoking during pregnancy, underweight, overweight/obese and diabetes. Others had increased prevalence or mean in higher average income areas including maternal age ≥ 35 and height.

**Table 6:** Maternal risk factors by census dissemination area income quintile

	Census dissemination area average income quintile					
	1 (lowest)	2	3	4	5 (highest)	2-sided p
n	11444	11510	11953	12163	11969	
			n (%)			
Smoking during pregnancy	690 (6)	807 (7)	815 (7)	698 (6)	468 (4)	<0.0001
Maternal age						
<20	269 (2)	211 (2)	201 (2)	144 (1)	102 (1)	<0.0001
20–35	8714 (76)	8667 (75)	8827 (74)	8738 (72)	7729 (65)	
≥ 35	2461 (22)	2632 (23)	2925 (24)	3281 (27)	4138 (35)	
Parity ≥ 1	5713 (50)	5643 (49)	5979 (50)	6119 (50)	6089 (51)	.0760
Pre-pregnancy BMI						
Underweight (<18.5)	1092 (10)	878 (8)	772 (6)	691 (6)	665 (6)	<0.0001
Normal weight (18.5–25)	7190 (63)	7167 (62)	7355 (62)	7727 (64)	8279 (69)	
Overweight (25–30)	2144 (19)	2278 (20)	2464 (21)	2471 (20)	2137 (18)	
Obese (≥30)	1018 (9)	1187 (10)	1362 (11)	1274 (10)	888 (7)	
Height (cm) – Mean (SD)	162 (7)	163 (7)	164 (7)	165 (7)	165 (7)	<0.0001
Diabetes/ Hypertension						
Neither	9388 (82)	9592 (83)	10061 (84)	10493 (86)	10629 (89)	<0.0001
Diabetes only	1444 (13)	1270 (11)	1188 (10)	984 (8)	729 (6)	
Hypertension only	463 (4)	512 (4)	563 (5)	577 (5)	538 (4)	
Diabetes & hypertension	149 (1)	136 (1)	141 (1)	109 (1)	73 (1)	
Oligohydramnios	308 (3)	281 (2)	264 (2)	258 (2)	272 (2)	0.0345
Placenta disorder	238 (2)	274 (2)	239 (2)	245 (2)	255 (2)	0.2528

Average income quintile ranges: quintile 1, $6,196–$24,444; quintile 2, $24,445–28,440; quintile 3, 28,441–$32,954; quintile 4, $32,987–$38,832; quintile 5, $38,838–$509,269. 2-sided *p* value is for chi squared test except for height, which is for analysis of variance (ANOVA).

Based on the results in Table 6, there appeared to be three variables that represented potential intervention points to reduce socioeconomic SGA inequality by average income: smoking during pregnancy, underweight and maternal height. Each of these variables was associated with higher odds of SGA after adjustment for other variables in Table 4. Additionally, each had significantly higher prevalence (or, for height, lower mean) among lower average income quintiles in Table 6. Although maternal height cannot be changed in adults, interventions could be designed to facilitate foetal and childhood growth to increase maternal height in the next generation [37]. For other variables, their prevalence of higher-risk values did not differ by average income (e.g., placental disorder). Higher diabetes prevalence was strongly associated with lower average income, but this factor was protective against SGA. Socioeconomic diabetes inequality is important to address in itself due to pathological effects on the offspring of diabetic mothers, but for this report we are focusing only on SGA.

We used a crude analysis of PAR to investigate the expected risk of SGA in each level of average income assuming the prevalence of smoking during pregnancy, underweight or bottom four quintiles of maternal height had been different. We first calculated the expected SGA risk for the situation in which the prevalence of each variable was equivalent to that found in the highest average income quintile. We then calculated the expected SGA risk for the situation in which the prevalence of high-risk values of the variables was 0% in each level of average income. The total reductions of SGA incidence for smoking during pregnancy and underweight in these scenarios (Table 7) were small, but much larger for maternal height. If all mothers had had height within the top quintile of this sample, we estimated that the incidence of SGA using the Canadian standard [24] would have been reduced from 7.2% to 3.9% (a 46% reduction). Relative socioeconomic SGA inequality would have been very slightly reduced from the observed 37% difference (8.9% versus 5.6%) to a 34% difference (4.7% versus 3.1%). But, absolute socioeconomic SGA inequality would have been reduced by 52% from 3.3% to 1.6%.

**Table 7:** Crude calculated risks of SGA after reduced smoking, underweight or shorter height prevalence

	Observed			SGA with reduced smoking prevalence *					
Model 1		SGA			SGA			SGA	
Average income quintile	% smokers	n	%	% smokers	n	%	% smokers	n	%
1 ($6,196–$24,444)	6.0	1018	8.9	3.9	1014	8.9	0.0	1006	8.8
2 ($24,444–$28,440)	7.0	916	8.0	3.9	915	7.9	0.0	913	7.9
3 ($28,440–$32,954)	6.8	876	7.3	3.9	871	7.3	0.0	864	7.2
4 ($32,986–$38,832)	5.7	752	6.2	3.9	742	6.1	0.0	719	5.9
5 ($38,837–$509,269)	3.9	675	5.6	3.9	675	5.6	0.0	660	5.5
Total	5.9	4237	7.2	3.9	4216	7.1	0.0	4162	7.0
	Observed			SGA with reduced underweight prevalence *					
Model 2		SGA			SGA			SGA	
Average income quintile	% underweight	n	%	% underweight	n	%	% underweight	n	%
1 ($6,196–$24,444)	9.5	1018	8.9	5.6	992	8.7	0.0	955	8.3
2 ($24,444–$28,440)	7.6	916	8.0	5.6	901	7.8	0.0	860	7.5
3 ($28,440–$32,954)	6.5	876	7.3	5.6	867	7.3	0.0	815	6.8
4 ($32,986–$38,832)	5.7	752	6.2	5.6	752	6.2	0.0	735	6.0
5 ($38,837–$509,269)	5.6	675	5.6	5.6	675	5.6	0.0	628	5.2
Total	6.9	4237	7.2	5.6	4186	7.1	0.0	3992	6.8
	Observed			SGA with reduced shorter height prevalence *					
Model 3		SGA			SGA			SGA	
Average income quintile	% shorter height**	n	%	% shorter height	n	%	% shorter height	n	%
1 ($6,196–$24,444)	85.9	1018	8.9	70.5	932	8.1	0.0	538	4.7
2 ($24,444–$28,440)	81.6	916	8.0	70.5	864	7.5	0.0	533	4.6
3 ($28,440–$32,954)	77.0	876	7.3	70.5	841	7.0	0.0	461	3.9
4 ($32,986–$38,832)	72.5	752	6.2	70.5	743	6.1	0.0	410	3.4
5 ($38,837–$509,269)	70.5	675	5.6	70.5	675	5.6	0.0	377	3.1
Total	77.4	4237	7.2	70.5	4055	6.9	0.0	2319	3.9

* SGA risks over 3.7 year period of study under scenarios of reduced maternal risk factors calculated by assuming crude risks of SGA would remain constant within joint levels of average income quintile and smoking (model 1), average income quintile and underweight (model 2), and average income quintile and shorter height (model 3).

** Shorter height defined as bottom four quintiles of height.

In a sensitivity analysis of potential selection bias, our model parameters were similar in models that did or did not contain BMI and height, so we used the model without BMI and height to compare model parameters among those who were included in and excluded from our analysis. The model parameters were similar among the 59,039 who were included in our analysis when compared to 20,805 (out of 23,306 total excluded for any of the reasons listed above in Results) who were excluded from the model due to missing values of height and BMI. The possibility of selection bias due to missing BMI and height is less than it would have been if the model parameters differed between these groups.

## Discussion

Our results show that DA average income is a useful indicator of area-level socioeconomic SGA inequality in Vancouver. Our finding of a statistically significant association of SGA by average income quintile is consistent with previous studies conducted using both area-level and individual-level income measures. In Canada, higher odds of preterm birth and SGA have been associated with lower average income even after adjustment for individual level characteristics [9,10]. However, one study found individual-level maternal education to be independently and more strongly associated with both birth outcomes than average income [10]. Unfortunately, our Registry data did not include individual-level maternal education, so we were unable to investigate this association. In the UK, it was found that there were higher odds of LBW in areas with greater area-level deprivation even after controlling for individual-level variables that were known risk factors for LBW [4,7,12]. Similarly, a study in the Netherlands found that neighbourhoods with lower median income had higher odds of SGA after adjustment for individual-level factors [2]. In contrast to these studies, we included smoking during pregnancy, which has previously been associated with SGA [15,25-27].

We found that mothers who lived in areas with lower average income were shorter and half of SGA cases were attributable to shorter height according to PAR%. It is important to consider some strong assumptions and unique characteristics of PAR% when interpreting this result. One assumption is that maternal height is a cause of SGA risk. Others are that additional risk factors are randomly distributed in the population and an intervention on maternal height would leave those distributions unchanged [38,39]. These latter two assumptions are unlikely to be true and are sufficient reason to avoid a strong interpretation of the PAR% as a prediction of what would result after intervention on height. Finally, we note that PAR% is a function not only of the magnitude of a factor’s association with the outcome, but also the prevalence of the factor in the population [38]. The large PAR% for height reflects, in part, our broad definition of shorter height, which resulted in high population prevalence. Because of these considerations, we interpret this result for height more qualitatively than quantitatively. Height is an important factor to consider in socioeconomic SGA inequality in greater Vancouver even if we do not know precisely the degree to which SGA risk (as defined in this analysis) would change in particular populations if average maternal height increased in future generations.

Despite these cautions of interpretation, our results may indicate a trans-generational aetiology for socioeconomic SGA inequalities in Vancouver. Based on evolutionary and human life-course theories, foetal growth is modulated in response to signals about the energy stores of the mother, such as maternal height (correlated with lean mass) and adipose tissue mass [40]. Maternal height and adipose tissue differ, though, in their plasticity in adulthood. Whereas adipose tissue mass can increase or decrease throughout life in response to energy intake, human growth is thought to be canalized by age two (although some catch-up in height can occur during adolescence) [41,42]. As such, adult height changes over generations rather than within individuals’ lives and is an indicator of multigenerational histories of conditions that facilitate growth [37]. Adult height at a population level is known to increase or decrease over generations in response environmental conditions, but is constrained by the height of the parental generation [37]. Our finding that mothers from areas with lower average income were shorter possibly indicates that historical variability between family lineages in access to favourable environmental conditions is mirrored within the social strata of Vancouver today. As such, the socioeconomic SGA inequality that we observed may be a consequence, in part, of socioeconomic differentials in historical access to environmental resources favouring growth. Another possible hypothesis is that taller height itself confers a competitive advantage in Vancouver’s society leading to greater upward social mobility.

However, our findings suggest that additional historical or contemporary causes of socioeconomic SGA inequality may also exist as evidenced by the strong negative association between current average income and SGA even after adjustment for maternal risk factors including height. Furthermore, average income had an adjusted PAR% of 14% for SGA, which was larger than that of any other risk factors except height and parity. Again, this result should be interpreted cautiously because the large PAR% for average income reflected, in part, our broad definition of lower average income. Future research should aim to elucidate the causal structures that bring about the association between SGA and contemporary area-level average income. Possibly, this association is the causal effect of fewer individual material resources among those living in areas of lower average income or might reflect poorer environmental quality in those areas. Although we found that area-level percentage employment and high school education were not associated with SGA when modelled together with area-level average income, it is important to note that these three area-level variables are geographically clustered in Vancouver and throughout Canada as evidenced by the principal components analysis upon which the INSPQ deprivation index is derived. Investigations of the causal structure of the association between SGA and average income should consider employment and education domains, as well. A possible reason that average area income, but not education, was associated with SGA might be that recent immigrants from other countries receive lower returns to years of schooling and experience than Canadians [43]. On the other hand, lower average income might be associated with other contemporary or historical causal phenomena leading to higher SGA risk today. In this analysis, we tested all of the known maternal risk factors for SGA that were available to us and found that none of them could explain relative socioeconomic SGA inequality. However, shorter maternal height explained about half of absolute socioeconomic SGA inequality. More research is needed to determine if small babies born to shorter mothers are at increased risk of adverse health outcomes and, if so, how stature could successfully be targeted to reduce socioeconomic SGA inequality. Current maternal height cannot be changed through intervention, but the height of the next generation may be influenced by interventions that affect foetal and childhood growth.

Our results may indirectly point to the importance and potential utility of focusing on social determinants of health in research and policy related to socioeconomic SGA inequality. Although individual risk factors for SGA (other than maternal height) might not be viable targets of intervention to reduce inequality when targeted one by one, our analysis did not investigate the potential benefits of targeting multiple risk factors at once. But if this is what would be needed, a potentially more efficient approach could be to identify and focus on the fundamental causes of health inequality that Phelan et al. [44] posit must exist because socioeconomic mortality inequality has persisted for centuries even though the major causes of mortality have shifted from infectious to chronic diseases. In this view, the inequality in health behaviours and comorbid disease states that we see in Vancouver today are not the fundamental causes of socioeconomic SGA inequality, but are instead the contemporary manifestations of deeper processes leading to health inequality. Frohlich et al. [45] suggest that income is a proxy for opportunities, resources and constraints, which are the fundamental causes of health inequality in populations. According to this view, the multiple forms of socioeconomic health inequality seen in Vancouver today might be addressed, for example, by focusing on equalizing the distribution of economic, cultural and social resources. Whatever the conceptual framework, more research and policy directed at the fundamental causes of health inequality may be a way forward given the results of our analysis.

Our analysis has several limitations. Although DAs are the smallest area for which aggregated income data are available from the census and so may be more homogenous than larger areas, without data on individual income we could not determine whether the association that we observed between average income and SGA was an individual- or area-level effect or both. We were also unable to include important variables previously associated with SGA, such as second-hand smoke exposure [46] and maternal race/ethnicity [8,15]. Another limitation was that our analysis used population data that were collected for clinical use rather than research. In particular, the sensitivity of classifying smoking during pregnancy might have been lowered due to its stigma and consequent underreporting by women to their healthcare providers. To the degree that smoking during pregnancy was under-reported, our calculated PAR% for SGA of smoking during pregnancy might also be underestimated.

An additional consideration is that although Perinatal Services BC has built-in validation rules and regular data quality checks [22], 61% of the sample was coded as non-smokers due to lack of data on smoking status during pregnancy. This recoding most likely resulted in some misclassification of smoking status. However, our estimate of 6% prevalence of maternal smoking is consistent with estimates found in an independent study [47]. Furthermore, a previous sensitivity study using randomly selected clinical records from the Registry found that recoding missing values as non-smokers led to 75% specificity and 98% sensitivity in smoking status classification overall [48]. While we cannot determine if misclassification was differential with regard to SGA status, the fact that smoking status was reported prior to birth (and observation of SGA status) makes differential misclassification of exposure less likely than if it had been reported after birth. For this reason, as well, we might have underestimated the importance of smoking in the pathogenesis of SGA overall as well as in its inequality between income groups.

## Conclusions

Census DA average income was a strong, linear indicator of socioeconomic SGA inequality in Vancouver. Our finding that mothers from DAs with lower average income were shorter possibly indicates that historical variability between family lineages in access to favourable environmental conditions is mirrored within the social strata of Vancouver today and contributes to socioeconomic SGA inequality. Another possible hypothesis is that taller height itself confers a competitive advantage in Vancouver’s society leading to greater upward social mobility. However, our findings suggest that additional historical or contemporary causes of socioeconomic SGA inequality may also exist as evidenced by the strong negative association between current average income and SGA even after adjustment for maternal risk factors including height. Overall reductions in SGA may result from interventions on the maternal risk factors investigated in this study. Absolute socioeconomic SGA inequality in future generations might possibly be reduced through intervention on foetal and childhood growth, but more research is needed to determine whether this is a viable or appropriate target for intervention.

## Abbreviations

BMI = Body-mass index; CMA = Census metropolitan area; DA = Canadian census dissemination area; INSPQ = Institut National de Santé Publique du Québec; LBW = Low birthweight; OR = Odds ratio; SGA = Small for gestational age.

## Competing interests

The authors declare that they have no competing interests in relation to the work described.

## Authors’ contributions

IH conducted the data analysis and drafted the manuscript. LHM, LC, PJ, BL, MH, AM, and RP provided important intellectual input on interpretation of the data and drafting the manuscript. LHM and LC also provided input on data analysis. SV designed the study, directed implementation and data analysis, and helped to draft the manuscript. All authors read and approved the final manuscript.

## Pre-publication history

The pre-publication history for this paper can be accessed here:

http://www.biomedcentral.com/1471-2458/12/333/prepub
